# Two New 4-Hydroxy-2-pyridone Alkaloids with Antimicrobial and Cytotoxic Activities from *Arthrinium* sp. GZWMJZ-606 Endophytic with *Houttuynia cordata* Thunb

**DOI:** 10.3390/molecules28052192

**Published:** 2023-02-27

**Authors:** Ying Yin, Dongyang Wang, Dan Wu, Wenwen He, Mingxing Zuo, Weiming Zhu, Yanchao Xu, Liping Wang

**Affiliations:** 1 State Key Laboratory of Functions and Applications of Medicinal Plants, Guizhou Medical University, Guiyang 550014, China; 2 Key Laboratory of Chemistry for Natural Products of Guizhou Province, Chinese Academy of Sciences, Guiyang 550014, China; 3 School of Pharmaceutical Sciences, Guizhou Medical University, Guiyang 550025, China; 4 Key Laboratory of Marine Drugs, Ministry of Education of China, School of Medicine and Pharmacy, Ocean University of China, Qingdao 266003, China

**Keywords:** pyridone alkaloids, *Arthrinium* sp., endophytic fungus, antibacterial, cytotoxicity

## Abstract

Two new 4-hydroxy-2-pyridone alkaloids furanpydone A and B (**1** and **2**), along with two known compounds N-hydroxyapiosporamide (**3**) and apiosporamide (**4**) were isolated from the endophytic fungus *Arthrinium* sp. GZWMJZ-606 in *Houttuynia cordata* Thunb. Furanpydone A and B had unusual 5-(7-oxabicyclo[2.2.1]heptane)-4-hydroxy-2-pyridone skeleton. Their structures including absolute configurations were determined on the basis of spectroscopic analysis, as well as the *X*-ray diffraction experiment. Compound **1** showed inhibitory activity against ten cancer cell lines (MKN-45, HCT116, K562, A549, DU145, SF126, A-375, 786O, 5637, and PATU8988T) with IC_50_ values from 4.35 to 9.72 µM. Compounds **1**, **3** and **4** showed moderate inhibitory effects against four Gram-positive strains (*Staphylococcus aureus*, methicillin-resistant *S. aureus*, *Bacillus Subtilis*, *Clostridium perfringens*) and one Gram-negative strain (*Ralstonia solanacarum*) with MIC values from 1.56 to 25 µM. However, compounds **1**–**4** showed no obvious inhibitory activity against two Gram-negative bacteria (*Escherichia coli* and *Pseudomonas aeruginosa*) and two pathogenic fungi (*Candida albicans* and *Candida glabrata*) at 50 µM. These results show that compounds **1**–**4** are expected to be developed as lead compounds for antibacterial or anti-tumor drugs.

## 1. Introduction

Since ricinine [1] and ilicicolin H [2] were found in the early 1970s, a series of 4-hydroxy-2-pyridinone alkaloids with diverse structures were reported [3,4,5,6]. These alkaloids were mainly isolated from plants and fungi and had good biological activity [7]. According to the position and type of substituents, C-3 was often replaced by alkanes (e.g., septoriamycin from *Septoria pistaciarum* [8]) or terpenes (e.g., tenelin from *Beauveria tenella* and *Beauveria bassiana* [9]), and C-5 was often replaced by phenyl (e.g., sambutoxin from *Fusarium sambucinum* [10]) or cyclohexyl (e.g., torrubiellone A-B from *Torrubiella* sp. [11]). There were also a small number of derivatives whose C-6 was replaced by alkanes (e.g., pyridomacrolidin from *Beauveria basiana* [12]). These kind of compounds usually have anti-inflammatory, antibacterial, cytotoxicity, antimalarial, antiviral, insecticidal, antioxidant, anti-fibrosis, neuroprotection, inhibition of protein tyrosine kinase, and so on [13,14,15,16], which have attracted widespread attention.

**Figure 1:**
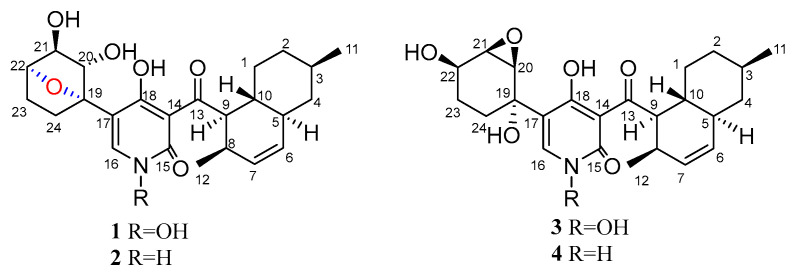
Structures of compounds **1**–**4**.

In the past few years, there were some new 4-hydroxy-2-pyridones discovered from fungi, such as (+)-didymellamide B, (±)-didymellamide E, (+)-*N*-hydroxyapiosporamide, and didymellamides F–H which were isolated from *Coniochaeta cephalothecoides* [17], and arthpyrones A and B with novel oxabicyclo[3.3.1]-nonane ring which were isolated from *Arthrinium arundinis* ZSDS1-F3 [18]. Three new 4-hydroxy-2-pyridone alkaloids citridones E–G with antibacterial activity were isolated from the endophytic fungus *Penicillium sumatrense* GZWMJZ-313 in our previous studies [19]. In order to obtain more compounds of this type from endophytic fungus, *Arthrinium* sp. GZWMJZ-606 was isolated from *Houttuynia cordata* Thunb. Further chemical investigation of this fungal strain led to the isolation of two new 4-hydroxy-2-pyridone derivatives (**1** and **2**) which we named furaprazone A and B (Figure 1), along with the known *N*-hydroxyapiosporamide (**3**) [18,20] and apiosporamide (**4**) [21]. Compounds **1** and **2** were the first reported compounds with 5-(7-oxabicyclo[2.2.1]heptane)-4-hydroxy-2-pyridone skeleton. Compared with the previously reported 1,2-epoxyhexane [17,21], 2-oxobicyclo [3.3.1] nonane [18] or benzene [22], 7-oxadicyclo [2.2.1] heptane can improve some biological activities [23,24,25]. Herein, the isolation, structure elucidation, the antimicrobial and cytotoxic activity of these compounds are described.

## 2. Results and Discussion

### Structure Elucidation

**Figure 2:**
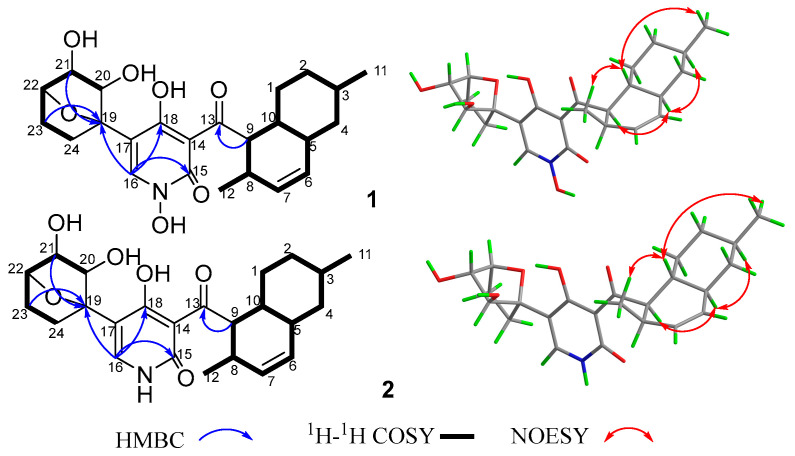
The key 2D NMR correlations of compounds **1** and **2**.

**Figure 3:**
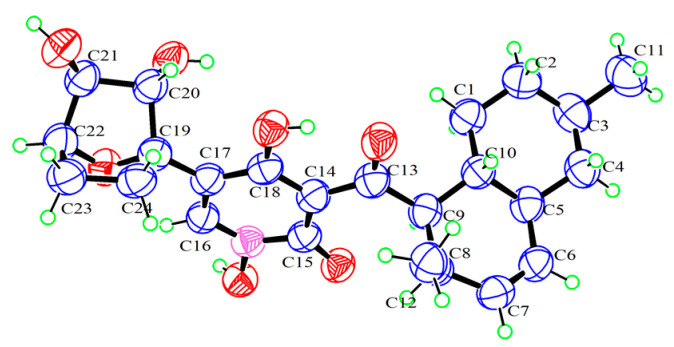
ORTEP drawing of **1**.

Compound **1** was obtained as a yellow crystal. The molecular formula was deduced as C_24_H_31_NO_7_ based on the HRESIMS ion peak at *m*/*z* 468.19861 [M + Na]^+^ (calcd. for C_24_H_31_NO_7_Na = 468.19927). Its IR (KBr) spectrum exhibited absorptions at 3434 cm^−1^ (hydroxy), 1649 cm^−1^ (carbonyl), and 1605/1552/1446 cm^−1^ (aromatic heterocycle). Compound **1** had the same molecular formula with *N*-hydroxyapiosporamide (**3**) and showed a high degree of similarity in UV absorption. The NMR spectra displayed two methyls, five sp^3^-methylenes, eight sp^3^-methines, three sp^2^-methines, one sp^3^-quaternary carbon, five sp^2^-quaternary carbons (including two carbonyls) (Table 1), which was also similar to those of compound **3**, especially for the important ^1^H NMR signals, such as two methyl groups at H_3_-11(*δ*_H_ 0.94) and H_3_-12 (*δ*_H_ 0.82), a single special hydrogen signal at H-16 (*δ*_H_ 7.93), two olefinic protons at H-6 (*δ*_H_ 5.41) and H-7 (*δ*_H_ 5.60). The above evidence suggested that compound **1** has a similar skeleton with compound **3**. The ^1^H-^1^H COSY correlations (Figure 2) from H_2_-1 (*δ*_H_ 0.88 and 1.95) to H-10 (*δ*_H_ 1.58), H-3 (*δ*_H_ 1.50) to H_3_-11, and H-8 (*δ*_H_ 2.85) to H-12 proved the existence of a decalin moiety. The relative configurations of this part were confirmed by the NOESY correlations (Figure 2) from H-10 to H_3_-11/H_3_-12 and H-5 (*δ*_H_ 1.83) to H-3/H-9 (*δ*_H_ 4.45), and indicated that compound **1** has the same decalin moiety as compound **3**. The ^1^H-^1^H COSY correlations from H-20 (*δ*_H_ 3.87) to H_2_-24 (*δ*_H_ 1.63 and 2.25) and the HMBC correlations (Figure 2) from H-21 (*δ*_H_ 4.00) and H_2_-23 (*δ*_H_ 1.70 and 2.25) to C-19 (*δ*_C_ 89.4) confirmed the presence of an oxygenated cyclohexane moiety in compound **1**. However, there was a large chemical shift difference between these two compounds at C-19/20/21/22 (*δ*_C_ 89.4, 82.1, 82.9, 78.6 for **1**; 70.4, 60.5, 57.7, 67.2 for **3**). Nevertheless, there is still one degree of unsaturation in the structure **1**, implying an oxygen bridge in this cyclohexane moiety, but the HMBC correlations cannot be used to confirm it. The key HMBC correlations from H-9 to C-13 (*δ*_C_ 211.4), H-16 to C-15 (*δ*_C_ 159.9)/C-18 (*δ*_C_ 173.2)/C-19 indicated that the decalin and hexane moieties substituted at C-13 and C-17 of the 4-hydroxy-2-pyridinone part. The crystal of compound **1** was fortunately acquired in methanol/water (*v/v*, 1:1) solution. The results of the X-ray (Figure 3) analysis (Flack parameter = −0.15 (11), CCDC: 2218951) confirmed an oxygen bridge between C-19 and C-22 forming the furan ring and led to the final determination of its absolute configuration as 3*R*, 5*S*, 8*R*, 9*R*, 10*R*, 19*S*, 20*S*, 21*S*, 22*S*. This novel 4-hydroxy-2-pyridone was named furanpydone A.

**Figure 4:**
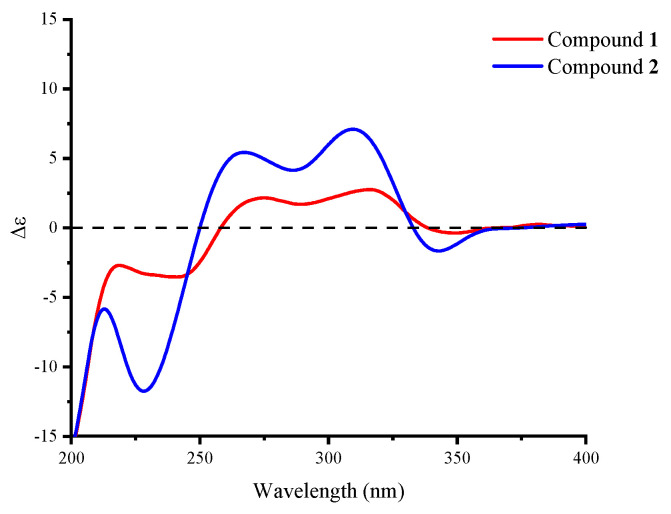
Experimental CD spectra of compounds **1** and **2**.

Compound **2** was obtained as a yellow powder. The molecular formula was deduced as C_24_H_31_O_6_N based on the HRESIMS peak at *m*/*z* 452.20319 ([M + Na]^+^, calcd. for 452.20436), which has ten degrees of unsaturation as furanpydone A (**1**), but one less oxygen atom than it. According to IR (KBr) spectrum data, they seem to have similar functional groups at 3445 cm^−1^ (hydroxy), 1652 cm^−1^ (carbonyl), and 1604/1557/1456 cm^−1^ (aromatic heterocycle). According to 1D NMR and HSQC data, compound **2** displayed two methyl (*δ*_H/C_ 0.74/17.9, 0.87/22.5), five sp^3^-methylene (*δ*_H/C_ 1.50 and 2.06/23.0; 0.81 and 1.83/29.6; 1.47 and 2.04/31.8; 0.96 and 1.67/35.1; 0.75 and 1.70/41.4), eight sp^3^-methines (*δ*_H/C_ 2.74/30.6, 1.46/32.6, 1.45/35.8, 1.75/41.4, 4.33/51.8, 4.30/76.6, 3.58/80.5, 3.78/81.2), three sp^2^-methines (*δ*_H/C_ 5.37/130.4, 5.57/131.7, 7.33/139.1), one sp^3^-quaternary carbon (*δ*_C_ 88.0), and five sp^2^-quaternary carbons (*δ*_C_ 106.7, 110.8, 161.8, 175.7, 209.6), which were extremely similar to compound **1** (Table 1 and Table S1) suggested the similar structure of these two compounds. The ^1^H-^1^H COSY correlations (Figure 2) from H_2_-1 (*δ*_H_ 0.81 and 1.83) to H-10 (*δ*_H_ 1.45), H-3 (*δ*_H_ 1.46) to H_3_-11 (*δ*_H_ 0.87), H-8 (*δ*_H_ 2.74) to H_3_-12 (*δ*_H_ 0.74), H-20 (*δ*_H_ 3.58) to H_2_-24 (*δ*_H_ 1.47 and 2.04), the key HMBC correlations (Figure 2) from H-9 (*δ*_H_ 4.33) to C-13 (*δ*_C_ 209.6), H-16 (*δ*_H_ 7.33) to C-15 (*δ*_C_ 161.8)/18 (*δ*_C_ 175.7)/19 (*δ*_C_ 88.0), H-21 (*δ*_H_ 3.78) to C-19 further confirmed that compound **2** and **1** have the same skeleton structure. Analysis of the NMR spectral data revealed that the chemical shift of C-15 (*δ*_C_ 161.8) moved to a lower field, which was similar to compound **4,** the key ^1^H-^1^H COSY correlation between H-16 and H-NH (*δ*_H_ 11.38) confirmed the absence of an N-hydroxy group in **2**. The key NOESY correlations from H-10 to H_3_-11/H_3_-12, H-5 (*δ*_H_ 1.75) to H-3 (*δ*_H_ 1.47)/H-9 (*δ*_H_ 4.33), as well as the same chemical shift for C-19/20/21/22/23/24 with furanpydone A (**1**) suggested that these two compounds had the same relative configuration. The similarity of electronic circular dichroism (ECD) curve of compound **2** (213 (−5.83), 228 (−11.74), 265 (+5.40), 310 (+7.09), 341 (−1.63) to **1** (217 (−2.73), 242 (−3.51), 270 (+2.00), 316 (+2.75), 343 (−0.27)) (Figure 4) along with the similar optical rotation values (**1**: −89.7, **2**: −80.0) indicated the same absolute configuration for **2** and **1**. This novel 4-hydroxy-2-pyridone was named furanpydone B.

**Figure 5:**
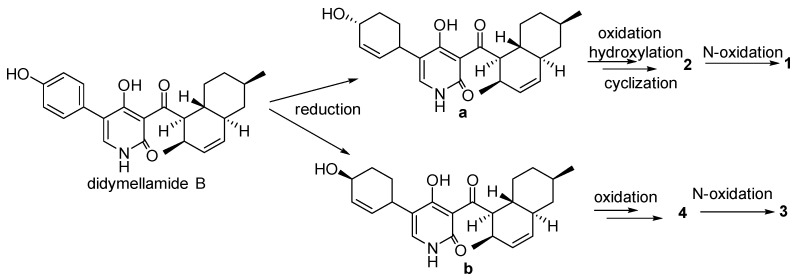
Proposed biosynthetic pathway for compounds **1**–**4**.

We propose a possible biosynthetic pathway for compounds **1**–**4**. Didymellamide B was the key intermediate in the biosynthesis of these compounds [18]. The intermediates **a** and **b** were obtained by reduction from didymellamide B. Compound **2** was obtained by oxidation, hydration and cyclization reaction from **a**, and compound **1** was syntheszed by further oxidation. Compound **4** was obtained by two oxidation reactions from **b**, and compound **3** was syntheszed by further oxidation. (Figure 5).

Compounds **1**–**4** were tested for their antimicrobial activities against nine pathogenic microorganisms. As shown in Table 2, compound **4** exhibited broad inhibitory activities against *Staphylococcus aureus*, methicillin-resistant *S. aureus* (MRSA), *Bacillus subtilis*, *Clostridium perfringens*, and *Ralstonia solanacarum* with the MIC values ranging from 1.56 to 6.25 µM. Compounds **1** and **3** showed moderate selective activities against *S. aureus* and MRSA with the MIC values of 12.5–25.0 µM. Compounds **1**–**4** showed no obvious inhibitory activity against two Gram-negative bacteria (*E. coli* and *P. aeruginosa*) and two pathogenic fungi (*C. albicans* and *C. glabrata*) at 50 µM. According to the results, it seems that the compounds with ternary epoxide showed better antibacterial activity than those with furan ring, but the effect of N-OH needs more research to determine.

The antiproliferative activities against 18 cancer cell lines and one normal cell line were assayed by the CCK-8 method. Compound **1** showed significant cytotoxicity against 10 cancer cell lines, compound **3** showed activities against HCT116 and 786-O cell lines (Table 3). The compounds with furan ring showed better antiproliferative activities than those with ternary epoxide. At the same time, nitrogen hydroxyl is the necessary group for maintaining the inhibitory activity.

## 3. Materials and Methods

### 3.1. General Experimental Procedures

The NMR spectra were recorded on Bruker Advance NEO 600 spectrometer (Bruker Corporation, Zurich, Switzerland) using TMS as an internal standard. MS analysis were carried out on Agilent 1100 instrument (Agilent Technologies, Santa Clara, CA, USA) and Thermo ultimate 3000/Q EXACTIVE FOCUS mass spectrometers (Thermo Scientific™, Waltham, MA, USA), respectively. Optical rotations were determined on Rudolph Autopol1 automatic polarimeter (Rudolph Research Analytical, Hackettstown, NJ, USA). UV spectra were detected on a Cary 60-UV-Vis spectrometer (Agilent Technologies, Santa Clara, CA, USA). IR spectra were determined on an iCAN 9 infrared spectrophotometer (Tianjin Nengpu Technology Co., Ltd, Tianjin, China) with KBr disks. X-ray data were generated using a Bruker Smart-1000 CCD (Bruker Corporation, Billerica, MA, USA) area detector diffractometer with graphite monochromatic Cu-Kα radiation. Column chromatography was performed on silica gel (200–300 mesh; Qingdao Puke Parting Materials Co., Ltd., Qingdao, China), Sephadex LH-20 gel (Amersham Biosciences, Uppsala, Sweden). HPLC separation was performed on HITACHI Primaide with an ODS-A column (YMC-pack ODS-A, 10 × 250 mm, 5 μm, 4 mL/min). Melting point instrument (SGW X-4).

### 3.2. Fungal Material

The endophytic fungus *Arthrinium* sp. GZWMJZ-606 was isolated from the leaves of *Houttuynia cordata* Thunb., which was collected from Longli, Guizhou, China. The leaves were treated with 75% alcohol for 30 s, and the residual alcohol was washed with sterile water. Then 1 g of fresh leaves was grinded into a pulp and 10 mL sterile water added. The suspension (100 μL) was deposited on a rice agar plate, which was prepared from rice powder (10 g), agar (18 g), and 1 L water containing chloramphenicol (0.3%) as a bacterial inhibitor, and incubated at 28 °C for 5 days. Monoclonal was selected and streaked to purity using the same agar medium. This strain was determined as *Arthrinium* sp. by the phylogenetic tree (Figure S1) of the ITS sequence (GenBank No. OP810989). The strain was deposited in our laboratory of Guizhou in 20% glycerol at −80 °C.

### 3.3. Fermentation and Extraction

The fungal strain GZWMJZ-606 was cultured on PDA at 28 °C for 3 days and then was cut into 100 × 1000 mL Erlenmeyer flasks, each containing a solid medium prepared from 100 g rice and 110 mL distilled water. These flasks were incubated at room temperature under static conditions for 40 days. The cultures were extracted three times by EtOAc (each 500 mL) and the combined EtOAc solutions were dried in vacuo to yield the extract (480.0 g).

### 3.4. Isolation and Purification

The EtOAc extract (480.0 g) was fractionated into 19 fractions (Fr.1–Fr.19) by chromatography on a silica gel column using step gradient elution of petroleum ether (PE)-EtOAc (*v*/*v*, 100:1–1:1) and CH_2_Cl_2_-MeOH (*v*/*v*, 20:1–1:1). Fr.17 (9.4 g) was further separated into 15 subfractions (Fr.17.1–Fr.17.15) by Sephadex LH-20 (CH_2_Cl_2_-MeOH, *v*/*v*, 1:1). Fr.17.11 (207.6 mg) was purified by semipreparative HPLC on an ODS-A column eluting with 60% MeCN-H_2_O containing 0.05% trifluoroacetic acid (TFA) to yield compound **1** (35.6 mg, *t*_R_ 11.1 min). Fr.17.14 (75.8 mg) was purified by semipreparative HPLC on an ODS-A column (60% MeCN-H_2_O containing 0.05% TFA) to yield compound **2** (6.8 mg, *t*_R_ 9.3 min). Fr.17.2 (830.5 mg) was further separated into 7 subfractions (Fr.17.2.1–Fr.17.2.7). Compound **4** (12.2 mg, *t*_R_ 10.1 min) was obtained from Fr.17.2.1 (57.1 mg) by semipreparative HPLC (55% MeCN-H_2_O containing 0.05% TFA). Fr.16 (1.6 g) was further separated into 6 subfractions (Fr.16.1–Fr.16.6), and Fr.16.4 (120.5 mg) was performed on a semipreparative ODS-A column (61% MeCN-H_2_O containing 0.05% TFA) to yield compound **3** (38.6 mg, *t*_R_ 8.4 min).

### 3.5. Physical Properties and Spectral Data of ***1***–***4***

Compound **1**: yellow crystal; m.p. 167.5–168.5 °C; ECD (1.12 *m*M, MeOH) *λ*max (Δ*ε*) 217 (−2.73), 242 (−3.51), 270 (+2.00), 316 (+2.75), 343 (−0.27) nm; [α${]}_{D}^{22}$−89.7 (*c* 0.58, MeOH); UV (MeOH) λ_max_ (log *ε*) 281 (0.75), 341 (0.72) nm; IR (KBr) *ν*_max_ 3434, 2913, 2953, 1649, 1605, 1446 cm^−1^; ^1^H NMR and ^13^C NMR data see Table 1 and Table S1 and Figures S3–S10; HRESIMS *m*/*z* 468.19861 [M + Na]^+^ (Figure S2), molecular formula: C_24_H_31_NO_7_.

X-ray crystallographic analyses of **1**: C_24_H_31_NO_7_·CH_3_OH, orthorhombic, *M* = 477.54, *a* = 7.6539 (3) Å, *b* = 14.5093 (6) Å, *c* = 21.7608 (11) Å, *α* = 90°, *β* = 90°, *γ* = 90°, *V* = 2416.60 (18) Å^3^, *T* = 150 K, space group *P*21 21 21, *Z* = 4, *μ* (Cu Kα) = 0.807 mm^−1^, 8074 reflections measured, 4534 independent reflections (*R_int_* = 0.019). The final *R_1_* values were 0.0724 (*I* > 2*σ* (*I*)). The final *wR* (*F*^2^) values were 0.1921 (*I* > 2*σ* (*I*)). The final *R_1_* values were 0.0767 (all data). The final *wR*(*F*^2^) values were 0.1991 (all data). The goodness of fit on *F*^2^ was 1.022. Flack parameter = −0.15 (11). CCDC: 2218951.

Compound **2**: yellow powder; ECD (1.17 *m*M, MeOH) *λ*max (Δ*ε*) 213 (−5.83), 228 (−11.74), 265 (+5.40), 310 (+7.09), 341 (−1.63) [α${]}_{D}^{22}$−80.0 (*c* 0.20, MeOH); UV (MeOH) λ_max_ (log *ε*) 235 (1.05), 270 (0.50), 338 (0.74) nm; IR (KBr) *ν*_max_ 3445, 2909, 1652, 1604, 1456 cm^−1^; ^1^H NMR and ^13^C NMR data see Table 1 and Figures S12–S17; HRESIMS *m*/*z* 452.20319 [M + Na]^+^ (Figure S11), molecular formula: C_24_H_31_NO_6_.

Compound **3**: yellow solid; the molecular formula is C_24_H_31_NO_7_ (*m*/*z* 444.1 [M − H]^−^) determined by ESIMS. [α${]}_{D}^{22}$−57.4 (*c* 2.3, MeOH); based on ^1^H NMR and ^13^C NMR data (Table 1, Figures S18 and S19) proved that compound **3** was N-hydroxyapiosporamide.

Compound **4**: faint yellow solid powder; the molecular formula is C_24_H_31_NO_6_ (*m*/*z* 452.5 [M + Na]^+^) determined by ESIMS. [α${]}_{D}^{22}$−32.2 (*c* 0.87, MeOH); based on ^1^H NMR and ^13^C NMR data (Table 1, Figures S20 and S21) proved that compound **4** was apiosporamide.

### 3.6. Antimicrobial Activities Assay

The isolated compounds were evaluated for antibacterial activity against pathogenic microorganisms including three Gram-negative strains (*Escherichia. coli* ATCC11775, *Pseudomonas aeruginosa* ATCC10145, *Ralstonia solanacarum* [26]), and four Gram-positive strains (*Staphylococcus aureus* ATCC6538, methicillin-resistant *S. aureus* ATCC43300 MRSA, *Clostridium perfringens* ATCC13124, and *Bacillus subtilis* ATCC6051), and two pathogenic fungi (*Candida albicans* ATCC10231 and *Candida glabrata* ATCC2001). The tested bacterial suspensions were incubated in Luria–Bertani (LB) medium and fungi in Mueller–Hinton agar (HMA) medium at 28 °C for 12 h and diluted to be 1 × 10^6^ CFU/mL by the same medium. Then, the DMSO solution of each compound was diluted into the corresponding concentration using the LB or MHA medium; 100 µL solution of compound was added into the first well of a 96-well plate and resulted the initial tested concentration of each compound to be 50 µmol/L (DMSO < 0.5‰ in each well) and the concentration of each compound to be 25 µmol/L (DMSO < 0.5‰ in each well) in the second well of a 96-well plate after then following this method in sequence, adding 100 µL microbial suspension into a 96-well plate. The ciprofloxacin and DMSO were used as the positive and negative controls, respectively. All experiments were repeated three times. MIC values were assessed by whether compounds can inhibit the growth of microorganisms [19].

### 3.7. Cytotoxic Activity Assay

Cell proliferation was measured with the CCK-8 method. By the dye of WST-8 (2-(2-methoxy-4-nitrophenyl)-3-(4-nitrophenyl)-5-(2,4disulfophenyl)-2H-etrazolium, monosodium salt) was reduced by dehydrogenase in cells to form a water-soluble tetrazolium salt product (formazan dye) with orange color. In the measurement, the amount of the formazan dye is proportional to the number of living cells. Finally, the cell viability can be estimated by recording the optical density (OD) of formazan dye at 450 nm using a microplate reader [27].

A cell suspension of 100 μL was dispensed (adherent cell viewed 5 × 10^4^/mL and suspension cell viewed 9 × 10^4^/mL) in 96-well plates. With doxorubicin hydrochloride as positive drug and DMSO as control, plates were pre-cultured for 24 h, followed by treatments with various concentrations of compound (eight concentration gradients were set for each sample for IC_50_ determination and three multiple holes were set for each concentration, *n* = 3). Keep the 96-well plates at 37 °C in an incubator with 5% CO_2_ for 72 h. After the aspiration of the old medium, the 10-fold diluted CCK-8 (100 μL) solution was added to each well of the plate, which was then incubated for another 3 h. An absorbance microplate reader was used to measure the absorbance at 450 nm. The optical density values (OD) of each well represented the survival/proliferation of cells. The toxicity is expressed by cell inhibition. The half inhibitory concentration (IC_50_) was defined as the concentration causing 50% inhibition, each group of data has 8 concentration gradient responses. The IC_50_ value is calculated by curve fitting using the software GraphPad Prism 8 (version 8.0.2, from GraphPad Software Inc., Boston, MA, USA), the experimental results are expressed in IC_50_ ± SD [28,29].

Cell inhibition rate = (OD_Control_ − OD_Drug_)/(OD_Control_ − OD_Blank_) × 100%.

The tested cell lines: A549: human lung cancer cells; MKN-45: human gastric cancer cells; HCT116: human colon cancer cells; K562: human chronic myeloid leukemia cells; DU145: human prostate cancer cells; SF126: human brain tumor cells; A-375: human malignant melanoma cells; MCF-7: human breast cancer cells; 786-O: human renal clear cell adenocarcinoma cells; PATU8988T: human pancreatic cancer cells; 5637: human bladder cancer cells; HeLa: human cervical cancer cells; TE-1: human esophageal cancer cells; GBC-SD: human gallbladder cancer cells; HepG2: human hepatoma cells; CAL-62: human thyroid cancer cells; HOS: human osteosarcoma cells; A-673: human rhabdomyosarcoma cells; L-02: human normal liver cells.

## 4. Conclusions

Two new 4-hydroxy-2-pyridone alkaloids were isolated from an endophytic fungus *Aspergillus* sp. GZWMJZ-606, which was obtained from *Houttuynia cordata* Thunb. Compounds **1** and **2** are the first example of 4-hydroxy-2-pyridone alkaloids possessing novel 7-oxidicyclo[2.2.1]heptane part. Compound **1** exhibited broad-spectrum cytotoxicity against 10 cancer cell lines with the IC_50_ values of 4.35–9.72 µM, and showed selective activities against *S*. *aureus* and MRSA *S*. *aureus* with MIC values of 12.5 µM. The discovery of novel 4-hydroxy-2-pyridinone alkaloids can provide a material basis for the discovery of potential drug molecules.

## Figures and Tables

**Table 1:** ^1^H (600 MHz) and ^13^C (150 MHz) NMR data of **1**–**4**.

Position	1 ^b^		2 ^a^		3 ^b^		4 ^b^	
	*δ*_C_	*δ*_H_ (*J* in Hz)	*δ*_C_	*δ*_H_ (*J* in Hz)	*δ*_C_	*δ*_H_ (*J* in Hz)	*δ*_C_	*δ*_H_ (*J* in Hz)
1	31.0, CH_2_	0.86–0.91, m 1.95, d (11.1)	29.6, CH_2_	0.77–0.85, m 1.83, dd (12.2, 3.0)	31.0, CH_2_	0.87–0.91, m 1.91–1.94, m	31.0, CH_2_	0.86–0.92, m 1.90–1.95, m
2	36.6, CH_2_	0.99–1.11, m 1.74, overlap	35.1, CH_2_	0.92–1.00, m 1.67, d (12.1)	36.6, CH_2_	1.00–1.09, m 1.73–1.77, overlap	36.6, CH_2_	1.00–1.08, m 1.73–1.78, overlap
3	34.4, CH	1.48–1.53, m	32.6, CH	1.43–1.49, overlap	34.3, CH	1.49–1.52, m	34.4, CH	1.48–1.53, m
4	43.2, CH_2_	0.80, t (12.2) 1.72–1.80, overlap	41.4, CH_2_	0.72–0.78, overlap 1.69–1.72, overlap	43.1, CH_2_	0.80, t (12.2) 1.73–1.77, overlap	43.2, CH_2_	0.79, t (12.2) 1.73–1.78, overlap
5	43.2, CH	1.83, “t” like (10.8)	41.4, CH	1.75, “t” like (10.1)	43.3, CH	1.81–1.85, overlap	43.2, CH	1.80–1.86, overlap
6	131.7, CH	5.41, d (9.7)	130.4, CH	5.37, d (9.9)	131.7, CH	5.42, d (9.9)	131.7, CH	5.41, d (9.8)
7	132.6, CH	5.58–5.63, m	131.7, CH	5.56–5.59, m	132.5, CH	5.58–5.62, m	132.6, CH	5.60, ddd (9.8, 4.5, 2.7)
8	32.3, CH	2.83–2.90, m	30.6, CH	2.71–2.76, m	32.3, CH	2.82–2.86, m	32.4, CH	2.80–2.86, m
9	54.5, CH	4.42–4.47, overlap	51.8, CH	4.33, dd (11.4, 5.7)	54.5, CH	4.45, dd (11.3, 5.7)	54.2, CH	4.43, dd (11.4, 5.8)
10	37.5, CH	1.56–1.60, overlap	35.8, CH	1.41–1.49, m	37.6, CH	1.56–1.60, m	37.6, CH	1.54–1.60, m
11	22.9, CH_3_	0.94, d (6.5)	22.5, CH_3_	0.87, d (6.5)	23.0, CH_3_	0.94, d (6.5)	22.9, CH_3_	0.93, d (6.5)
12	18.4, CH_3_	0.82, d (7.0)	17.9, CH_3_	0.74, d (7.3)	18.4, CH_3_	0.83, d (7.3)	18.4, CH_3_	0.83, d (7.2)
13	211.4, C		209.6, C		211.7, C		212.0, C	
14	108.2, C		106.7, C		108.6, C		108.8, C	
15	159.9, C		161.8, C		159.6, C		163.9, C	
16	139.5, CH	7.93, s	139.1, CH	7.33, d (6.0)	140.0, CH	8.04, s	139.9, CH	7.58, s
17	111.0, C		110.8, C		114.5, C		116.6, C	
18	173.2, C		175.7, C		175.6, C		179.3, C	
19	89.4, C		88.0, C		70.4, C		70.4, C	
20	82.1, CH	3.87, brs	80.5, CH	3.58, dd (6.1, 1.4)	60.5, CH	3.66, d (3.7)	60.5, CH	3.64, “t” like (2.1)
21	82.9, CH	4.00, d (4.3)	81.2, CH	3.78, “t” like (4.7)	57.7, CH	3.43, “t” like (3.3)	57.6, CH	3.42, “t” like (3.3)
22	78.6, CH	4.42–4.47, overlap	76.6, CH	4.30, “t” like (5.1)	67.2, CH	4.12–4.14, m	67.2, CH	4.13, ddd (8.6, 5.7, 2.8)
23	24.1, CH_2_	1.68–1.73, overlap 2.20–2.27, overlap	23.0, CH_2_	1.45–1.54, m 2.02–2.10, overlap	25.7, CH_2_	1.34–1.38, m 1.81–1.85, overlap	25.8, CH_2_	1.32–1.38, m 1.80–1.86, overlap
24	33.1, CH_2_	1.60–1.65, overlap 2.20–2.27, overlap	31.8, CH_2_	1.45–1.49, overlap 2.01–2.08, overlap	31.8, CH_2_	1.70, ddd (13.9, 10.2, 2.8) 2.26, ddd (14.4, 8.4, 2.5)	31.6, CH_2_	1.70, ddd (13.5, 10.4, 2.5) 2.22, dd (13.3, 8.6)
20-OH				4.77, d (6.1)				
21-OH				5.31, d (4.7)				
-NH				11.38, brs				

^a^ measured in DMSO-*d*_6_, ^b^ measured in methanol-*d*_4_ solvent.

**Table 2:** Antimicrobial activity of **1**–**4** (MIC, µM), *n* = 3.

Pathogenic Bacteria	1	2	3	4	Positive Drug
*E. coli* ATCC 11775	>50	>50	>50	>50	0.10 *
*P. aeruginosa* ATCC 10145	>50	>50	>50	>50	1.56 *
*S. aureus* ATCC6538	12.5	>50	12.5	6.25	0.20 *
MRSA ATCC 43300	12.5	>50	25.0	6.25	0.38 *
*B. subtilis* ATCC 6051	>50	>50	>50	1.56	6.25 *
*C. perfringens* ATCC 13124	>50	>50	>50	3.13	0.047 *
*R. solanacarum*	>50	>50	>50	6.25	3.12 *
*C. albicans* ATCC10231	>50	>50	>50	>50	3.13 ^#^
*C. glabrata* ATCC2001	>50	>50	>50	>50	3.13 ^#^

* the positive drug is ciprofloxacin, ^#^ the positive drug is amphotericin B.

**Table 3:** Cytotoxic activity (µM, IC_50_ ± SD), *n* = 3.

Cell Line	1	2	3	4	Dox
A549	6.47 ± 0.31	>10	>10	>10	0.849 ± 0.013
MKN-45	5.41 ± 0.09	>10	>10	>10	0.307 ± 0.005
HCT116	5.64 ± 0.05	>10	6.09 ± 0.02	>10	0.121 ± 0.005
K562	9.22 ± 0.93	>10	>10	>10	0.948 ± 0.058
DU145	9.01 ± 0.07	>10	>10	>10	0.189 ± 0.003
SF126	9.72 ± 0.46	>10	>10	>10	0.164 ± 0.016
A-375	7.16 ± 0.17	>10	>10	>10	0.064 ± 0.003
786-O	5.93 ± 0.13	>10	9.13 ± 0.48	>10	0.726 ± 0.028
PATU8988T	6.46 ± 0.09	>10	>10	>10	0.167 ± 0.012
5637	4.35 ± 0.08	>10	>10	>10	0.185 ± 0.002
HeLa	>10	>10	>10	>10	0.177 ± 0.006
TE-1	>10	>10	>10	>10	0.240 ± 0.030
GBC-SD	>10	>10	>10	>10	0.592 ± 0.069
MCF-7	>10	>10	>10	>10	0.966 ± 0.011
HepG2	>10	>10	>10	>10	0.619 ± 0.054
CAL-62	>10	>10	>10	>10	0.277 ± 0.019
HOS	>10	>10	>10	>10	0.090 ± 0.013
A-673	>10	>10	>10	>10	0.380 ± 0.030
L-02	7.09 ± 0.10	>10	9.70 ± 0.06	>10	0.243 ± 0.005

## Data Availability

Not available.

## Supplementary Materials

The following supporting information can be downloaded at: www.mdpi.com/article/10.3390/molecules28052192/s1, ITS1 gene sequences of *Arthrinium* sp. GZWMJZ-606; Figure S1: Species identification of endophytic fungi strain; Table S1: ^1^H (600 MHz) and ^13^C (150 MHz) NMR data of compound **1** DMSO-*d*_6_; Figure S2: HRESIMS spectrum of **1**; Figure S3: ^1^H NMR spectrum (600 MHz, Methanol-*d*_4_) of **1**; Figure S4: ^13^C NMR spectrum (150 MHz, Methanol-*d*_4_) s of **1**; Figure S5: HSQC spectrum (Methanol-*d*_4_) of **1**; Figure S6: HMBC spectrum (Methanol-*d*_4_) of **1**; Figure S7: ^1^H-^1^H COSY spectrum (Methanol-*d*_4_) of **1**; Figure S8: NOESY spectrum (Methanol-*d*_4_) of **1**; Figure S9: ^1^H NMR spectrum (600 MHz, DMSO-*d*_6_) of **1**; Figure S10: ^13^C NMR spectrum (150 MHz, DMSO-*d*_6_) of **1**; Figure S11: HRESIMS spectrum of **2**; Figure S12: ^1^H NMR spectrum (600 MHz, DMSO-*d*_6_) of **2**; Figure S13: ^13^C NMR spectrum (150 MHz, DMSO-*d*_6_) of **2**; Figure S14: HSQC spectrum (DMSO-*d*_6_) of **2**; Figure S15: HMBC spectrum (DMSO-*d*_6_) of **2**; Figure S16: ^1^H-^1^H COSY spectrum (DMSO-*d*_6_) of **2**; Figure S17: NOESY spectrum (DMSO-*d*_6_) of **2**; Figure S18: ^1^H NMR spectrum (600 MHz, Methanol-*d*_4_) of **3**; Figure S19: ^13^C NMR spectrum (150 MHz, Methanol-*d*_4_) s of **3**; Figure S20: ^1^H NMR spectrum (600 MHz, Methanol-*d*_4_) of **4**; Figure S21: ^13^C NMR spectrum (150 MHz, Methanol-*d*_4_) of **4**.

## Sample Availability

Samples of the compounds are available from the authors.
