# Supplementary material for: Two New 4-Hydroxy-2-pyridone Alkaloids with Antimicrobial and Cytotoxic Activities from Arthrinium sp. GZWMJZ-606 Endophytic with Houttuynia cordata Thunb
Source: Molecules. 2023 Feb 27;28(5):2192. doi: 10.3390/molecules28052192 (PMC10005160; doi:10.3390/molecules28052192)

molecules

Supplementary Materials

Two New 4-Hydroxy-2-pyridone Alkaloids with Antimicrobial and Cytotoxic Activities from *Arthrinium* sp. GZWMJZ-606 Endophytic with *Houttuynia cordata* Thunb.

Ying Yin[1,2,3,†], Dongyang Wang[1,2,†], Dan Wu[1,2], Wenwen He[1,2], Mingxing Zuo[1,2], Weiming Zhu[1,4], Yanchao Xu[1,3,*] and Liping Wang[1,2,3,*]

1 State Key Laboratory of Functions and Applications of Medicinal Plants, Guizhou Medical University, Guiyang 550014, China
2 Key Laboratory of Chemistry for Natural Products of Guizhou Province, Chinese Academy of Sciences, Guiyang 550014, China
3 School of Pharmaceutical Sciences, Guizhou Medical University, Guiyang 550025, China
4 Key Laboratory of Marine Drugs, Ministry of Education of China, School of Medicine and Pharmacy, Ocean University of China, Qingdao 266003, China
* Correspondence: xuyanchao@gmc.edu.cn (Y.X.), lipingw2006@163.com (L.W.)
† These authors contributed equally to the work.

Contents of Supporting Information

ITS1 gene sequences of *Arthrinium* sp. GZWMJZ-606. 3

Figure S1. Species identification of endophytic fungi strain 3

Table S1. ^{1}H (600 MHz) and ^{13}C (150 MHz) NMR data of **1** in DMSO-d_6 4

Figure S2. HRESIMS spectrum of **1** 5

Figure S3. ^{1}H NMR spectrum (600MHz, Methanol-d_4) of **1** 5

Figure S4. ^{13}C NMR spectrum (150 MHz, Methanol-d_4) of **1** 6

Figure S5. HSQC spectrum (Methanol-d_4) of **1** 6

Figure S6. HMBC spectrum (Methanol-d_4) of **1** 7

Figure S7. ^{1}H-^{1}H COSY spectrum (Methanol-d_4) of **1** 7

Figure S8. NOESY spectrum (Methanol-d_4) of **1** 8

Figure S9. ^{1}H NMR spectrum (600MHz, DMSO-d_6) of **1** 8

Figure S10. ^{13}C NMR spectrum (150 MHz, DMSO-d_6) of **1** 9

Figure S11. HRESIMS spectrum of **2** 9

Figure S12. ^{1}H NMR spectrum (600MHz, DMSO-d_6) of **2** 10

Figure S13. ^{13}C NMR spectrum (150 MHz, DMSO-d_6) of **2** 10

Figure S14. HSQC spectrum (DMSO-d_6) of **2** 11

Figure S15. HMBC spectrum (DMSO-d_6) of **2** 11

Figure S16. ^{1}H-^{1}H COSY spectrum (DMSO-d_6) of **2** 12

Figure S17. NOESY spectrum (DMSO-d_6) of **2** 12

Figure S18. ^{1}H NMR spectrum (600MHz, Methanol-d_4) of **3** 13

Figure S19. ^{13}C NMR spectrum (150 MHz, Methanol-d_4) of **3** 13

Figure S20. ^{1}H NMR spectrum (600MHz, Methanol-d_4) of **4** 14

Figure S21. ^{13}C NMR spectrum (150 MHz, Methanol-d_4) of **4** 14

ITS sequence (GenBank accession no. OP810989):
ATGCGGAGGGATCATTACAGAGTTATACAACTCCCATACCATCTGTTAACCTACCCAGTTA
TGCCTCGGCGTAAGCTCGGTTGGAGGCACCTGCAGCTACCCTGTAGTTGCGGACTGCCAA
CTCCAGCCGCGGCCCGCCGGCGGTACACTAAACTCTGTTTTATTTTATATTCTGAGCGTCTT
ATTTTAATAAGTTAAAACTTTCAACAACGGATCTCTTGGTTCTGGCATCGATGAAGAACGC
AGCGAAATGCGATAAGTAATGTGAATTGCAGAATTCAGTGAATCATCGAATCTTTGAACG
CACATTGCGCCCATCAGTATTCTGGTGGGCATGCCTGTTCGAGCGTCATTTCAACCCTTAA
GCCTAGCTTAGTGTTGGGAATCTGCTGTACTGCAGTTCCTTAAAGACAGTGGCGGAGCGG
CGGTAGTCCTCTGAGCGTAGTAATTTATTTCTCGCTTTTGTCAGGCTCTGTCCTCCCGCCAT
AAAACCCCCAATTTTTTAGTGGTTGACCTCGGATCAGGTAGGAATACCCGCTGAACTTAA
GCATATCAAAACGGGGGAAGGAAA

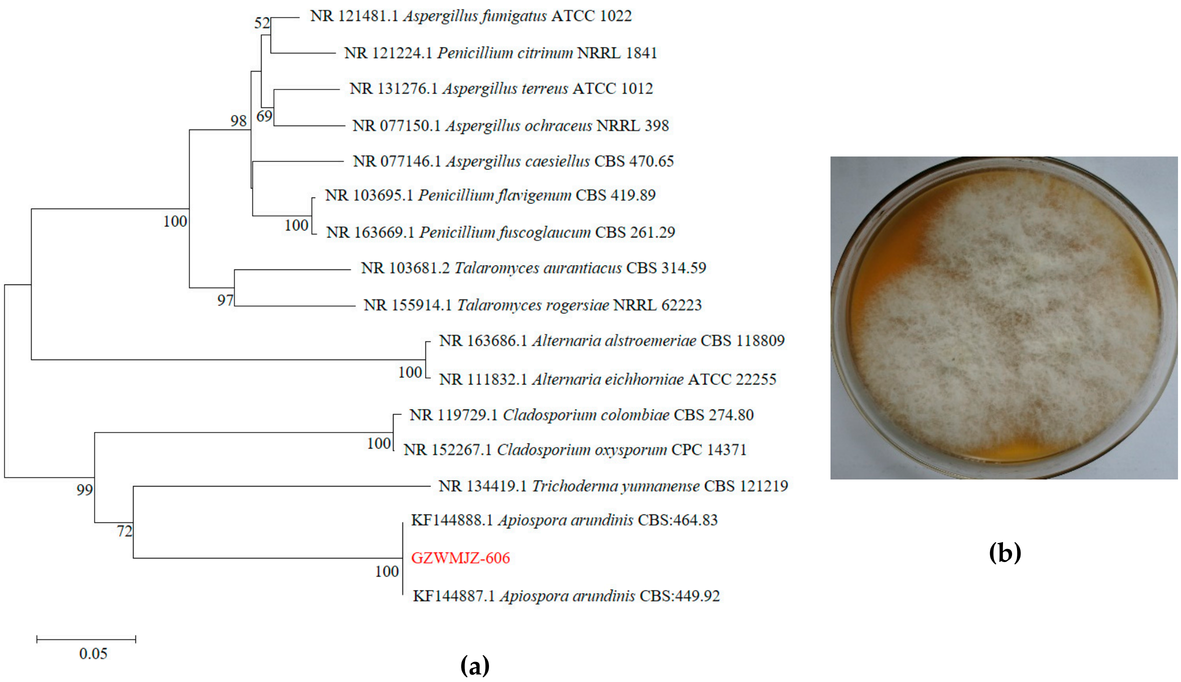

(a)

Figure S1. Species identification of endophytic fungi strain from *Houttuynia cordata*. **(a)** Phylogenetic tree of fungal strain *Arthrinium* sp. GZWMJZ-606 based on ITS sequences. **(b)** Colony of *Arthrinium* sp. GZWMJZ-606 was observed by growth on a PDA plate for 3 days.

Table S1. ^{1}H (600 MHz) and ^{13}C (150 MHz) NMR data of **1** in DMSO-d_6

Position	**1** (DMSO-d_6)	
	δ_C	δ_H (*J* in Hz)
1	29.5, CH_2	0.78-0.86, m<br>1.83, dd (12.4, 2.9)
2	35.0, CH_2	0.93-1.02, m<br>1.67, d (12.0)
3	32.5, CH	1.47-1.51, overlap
4	41.4, CH_2	0.72-0.78, overlap<br>1.70-1.74, overlap
5	41.3, CH	1.77, ''t'' like (10.5)
6	130.4, CH	5.39, d (9.8)
7	131.4, CH	5.57-5.63, m
8	30.6, CH	2.70-2.78, m
9	52.2, CH	4.35, dd (11.4, 5.7)
10	35.7, CH	1.42-1.47, overlap
11	22.4, CH_3	0.88, d (6.5)
12	17.8, CH_3	0.75, d (7.1)
13	209.4, C	
14	106.6, C	
15	157.5, C	
16	138.8, CH	7.71, s
17	108.8, C	
18	171.9, C	
19	87.6, C	
20	80.4, CH	3.59, brs
21	81.0, CH	3.79, d (4.8)
22	76.6, CH	4.32, ''t'' like (4.7)
23	22.9, CH_2	1.51-1.54, overlap<br>2.06-2.10, overlap
24	31.6, CH_2	1.47-1.51, overlap<br>1.99-2.06, overlap
20-OH		4.83, brs
21-OH		5.33, brs
N-OH		11.66, s

Figure S2. HRESIMS spectrum of **1**

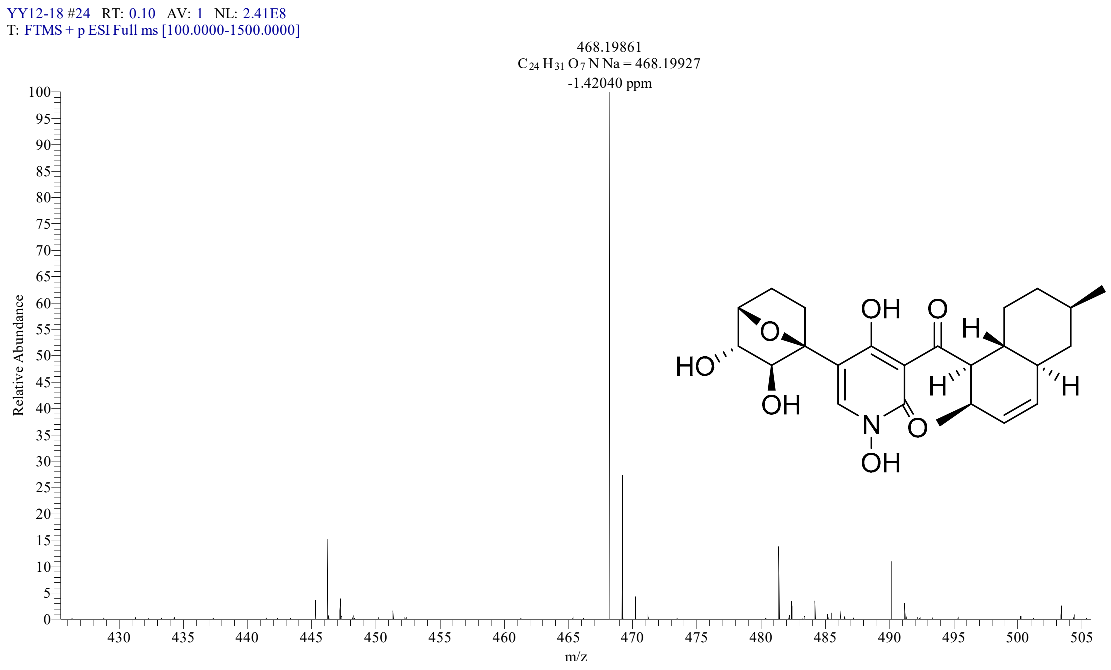

Figure S3. ^{1}H NMR (600MHz, Methanol-d_4) of **1**

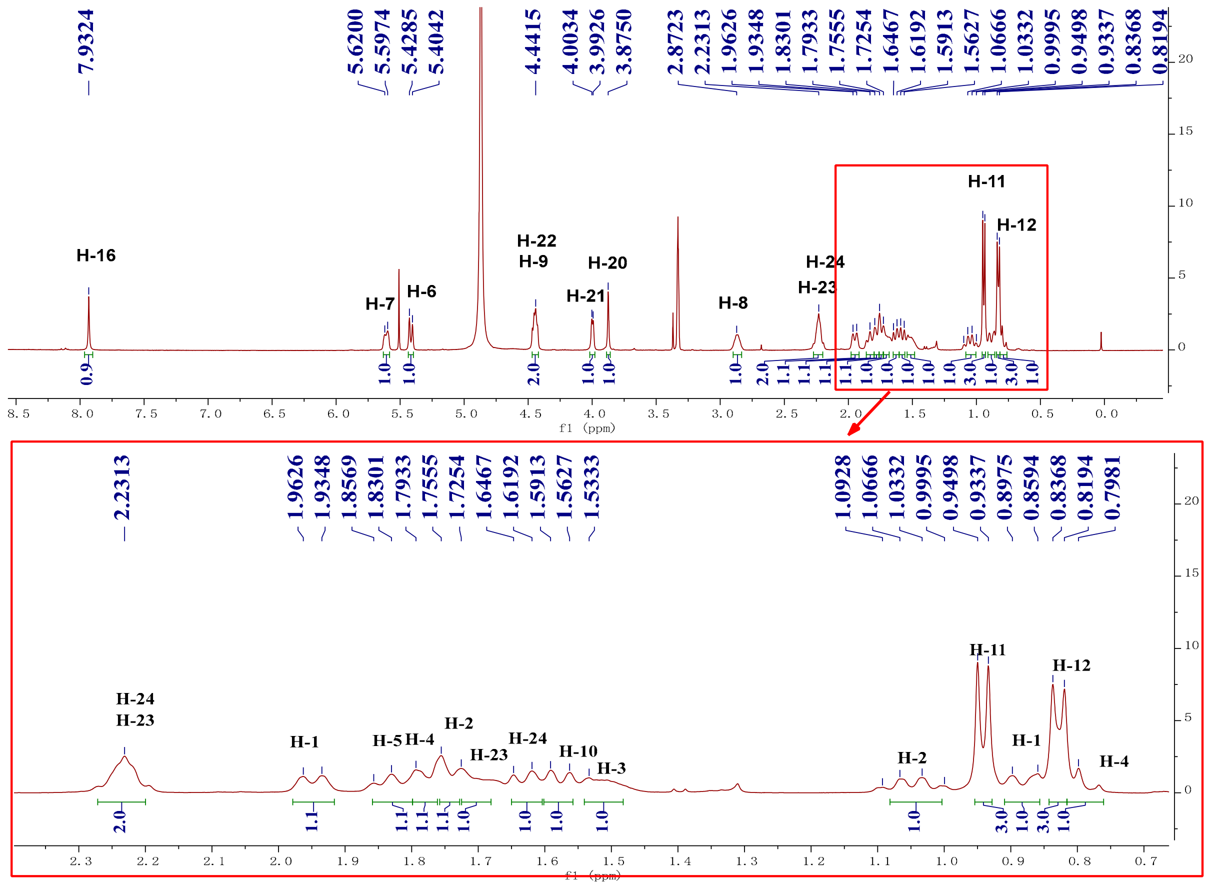

Figure S4. ^{13}C NMR (150 MHz, Methanol-d_4) of **1**

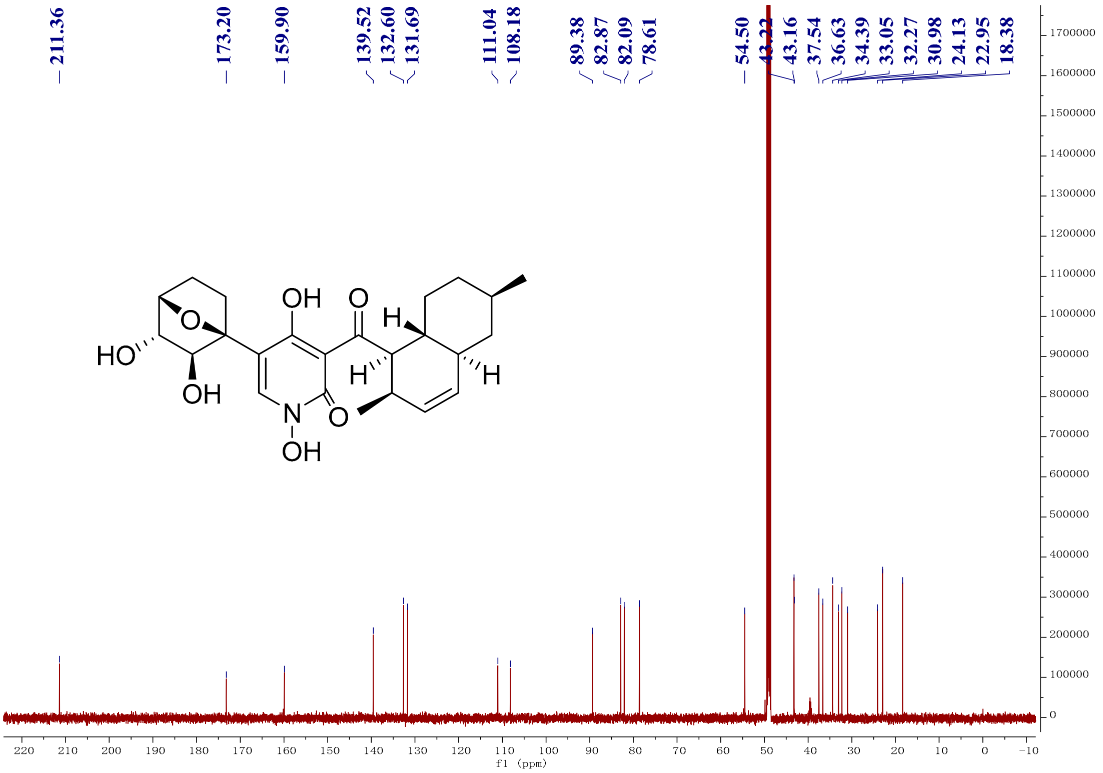

Figure S5. HSQC spectrum (Methanol-d_4) of **1**

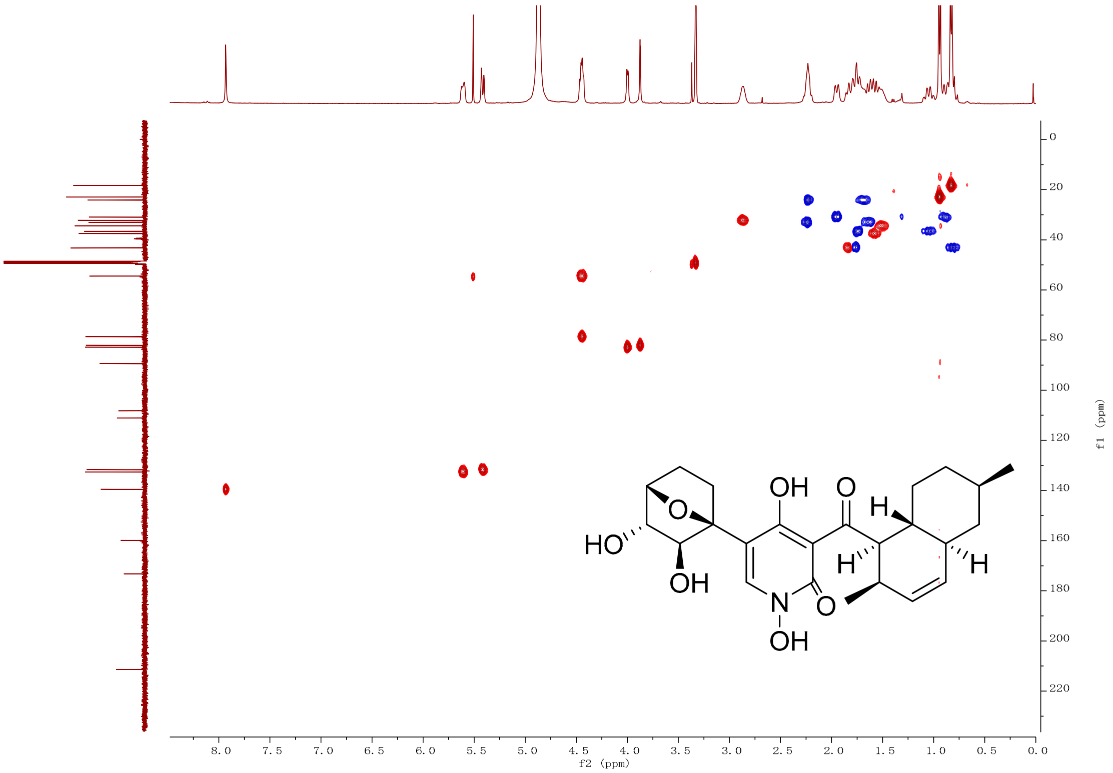

Figure S6. HMBC spectrum (Methanol-d_4) of **1**

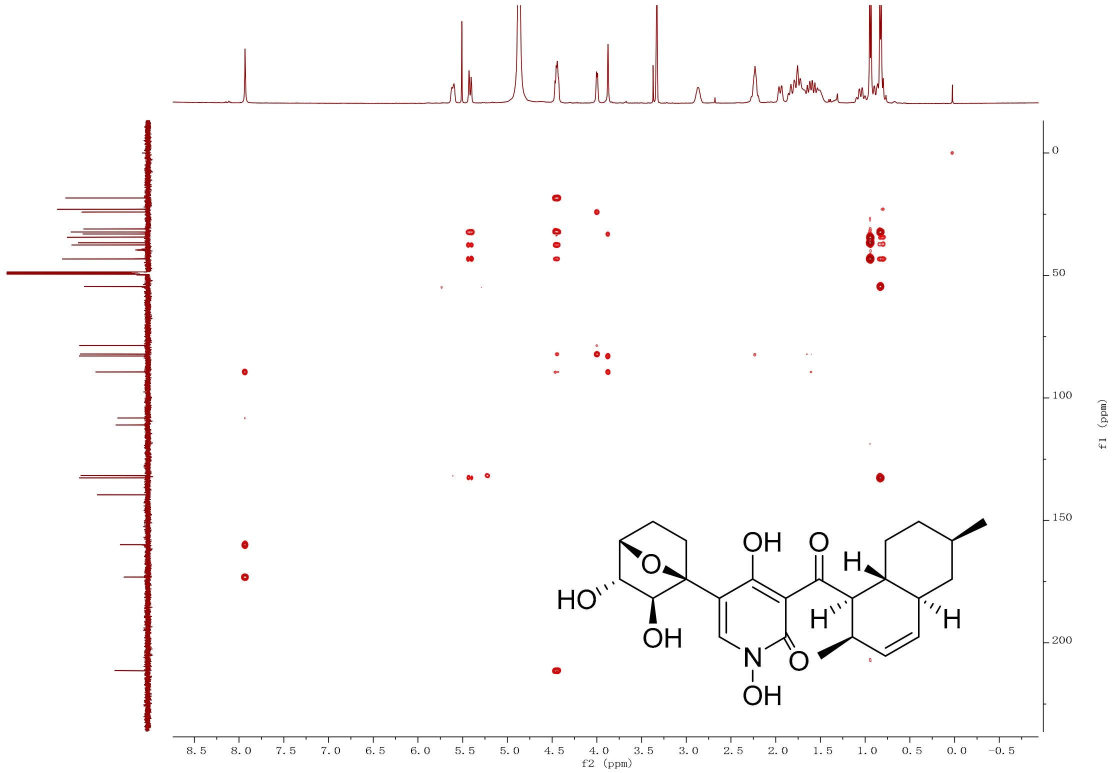

Figure S7. ^{1}H-^{1}H COSY spectrum (Methanol-d_4) of **1**

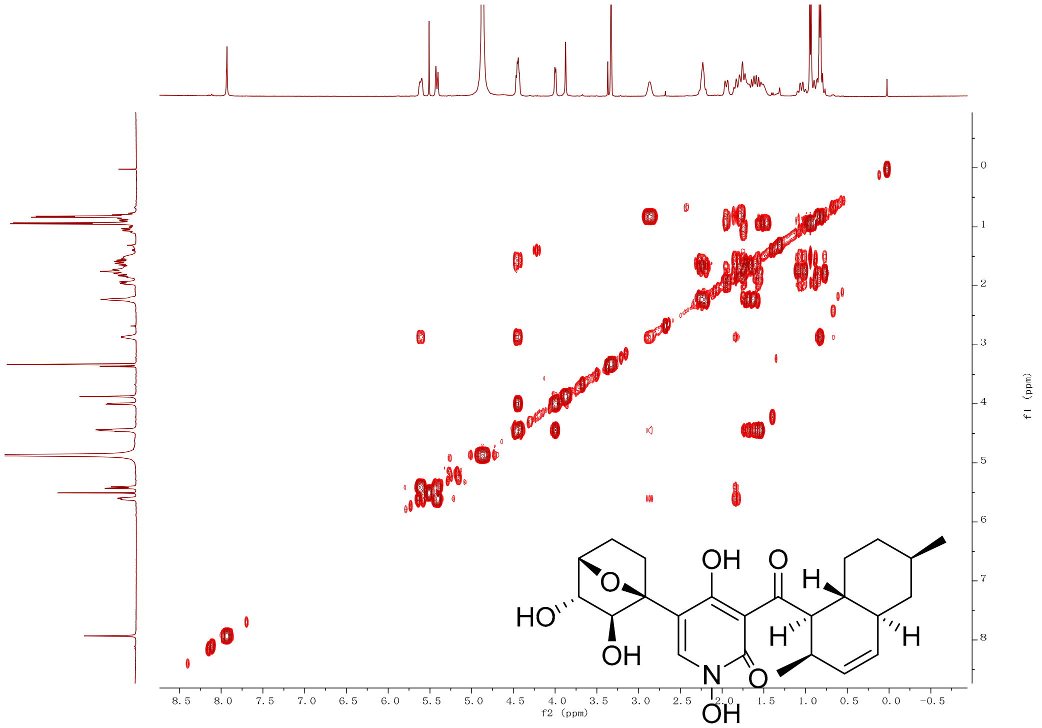

Figure S8. NOESY spectrum (Methanol-d_4) of **1**

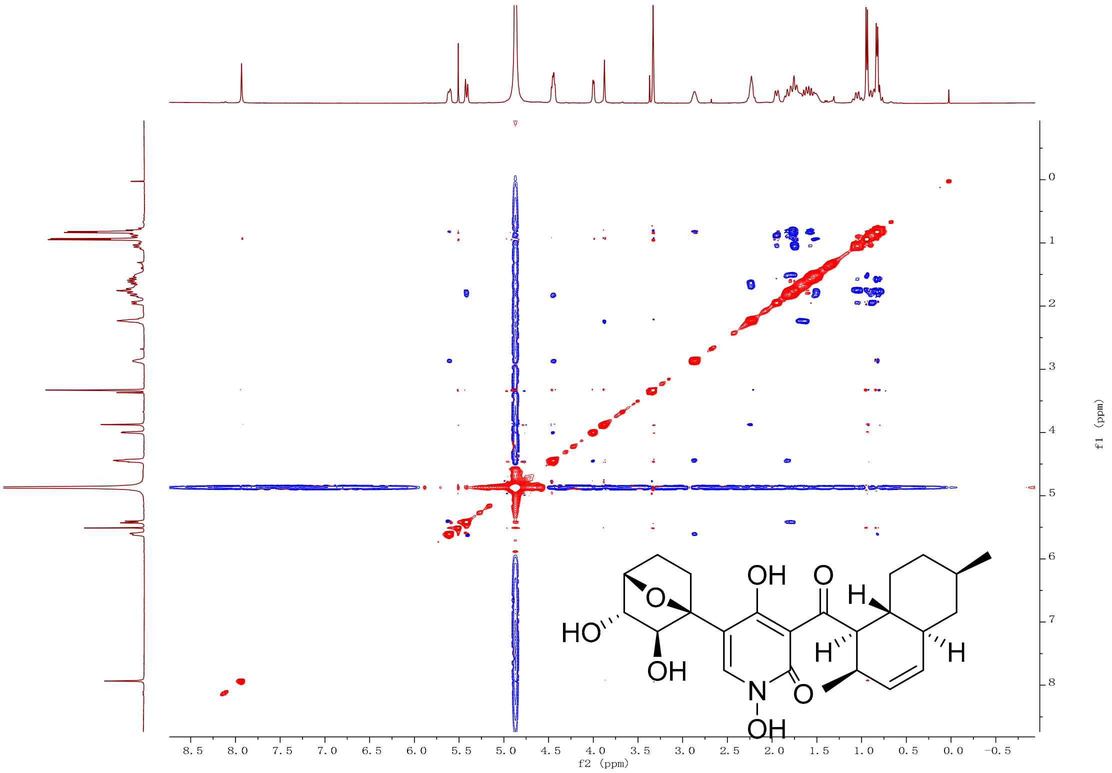

Figure S9. ^{1}H NMR (600MHz, DMSO-d_6) of **1**

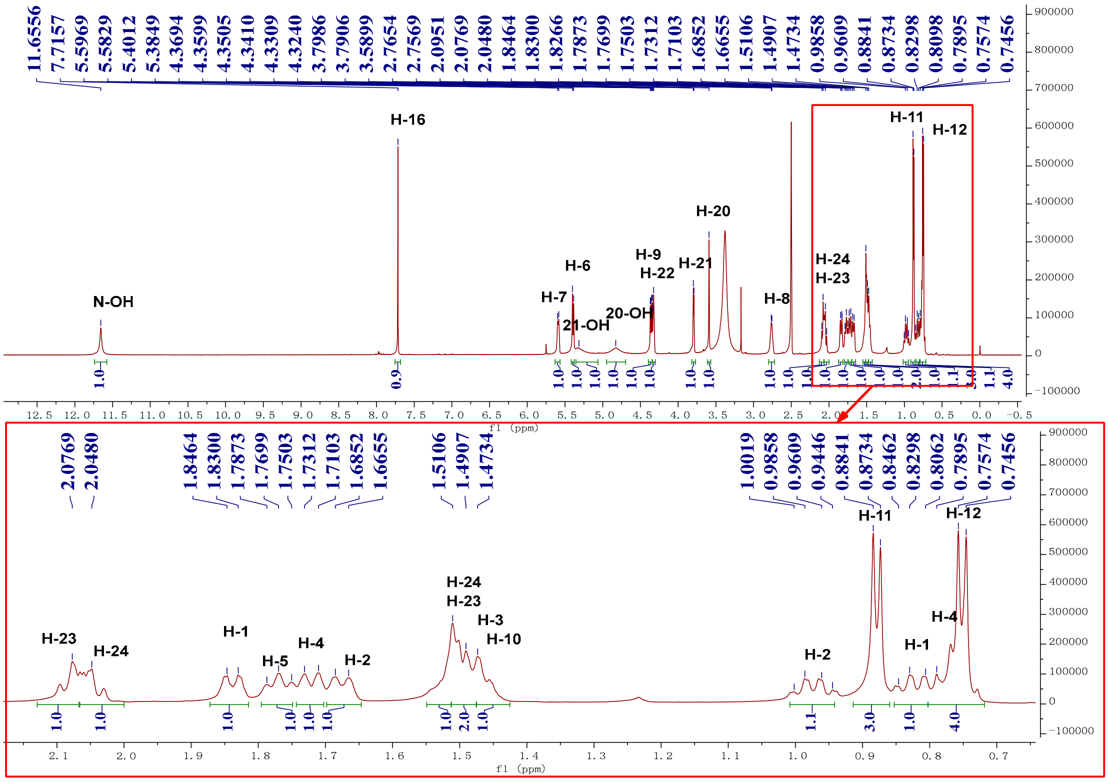

Figure S10. ^{13}C NMR (150 MHz, DMSO-d_6) of **1**

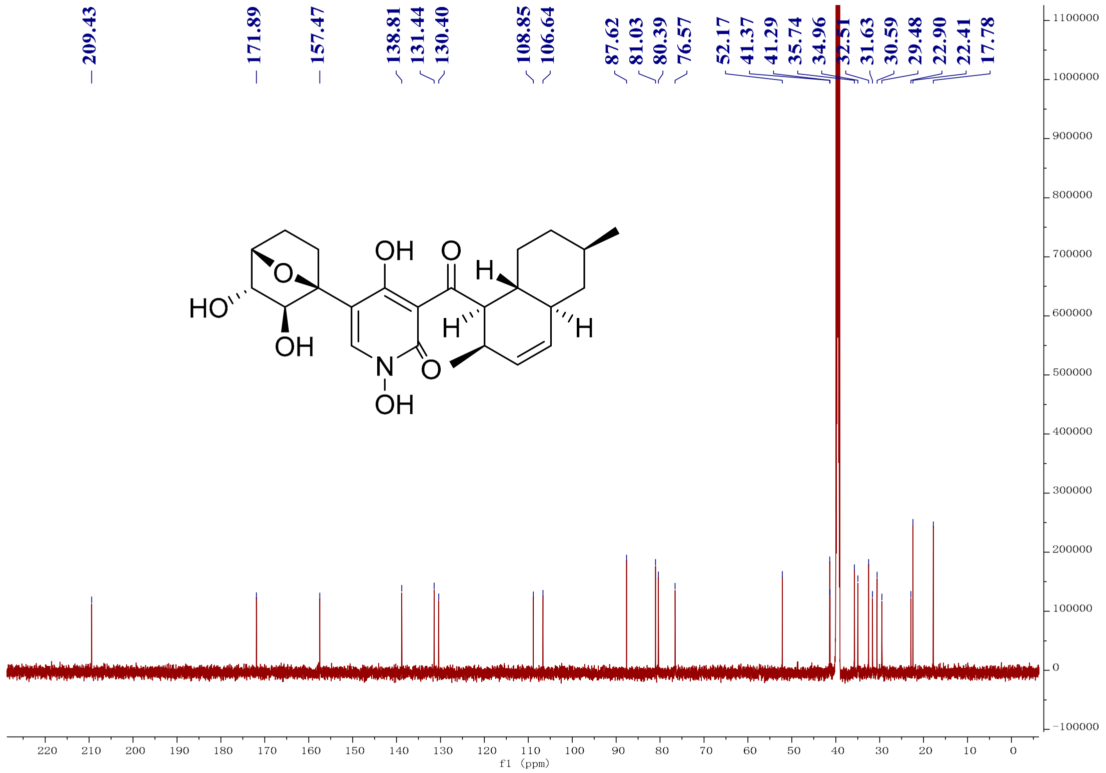

Figure S11. HRESIMS spectrum of **2**

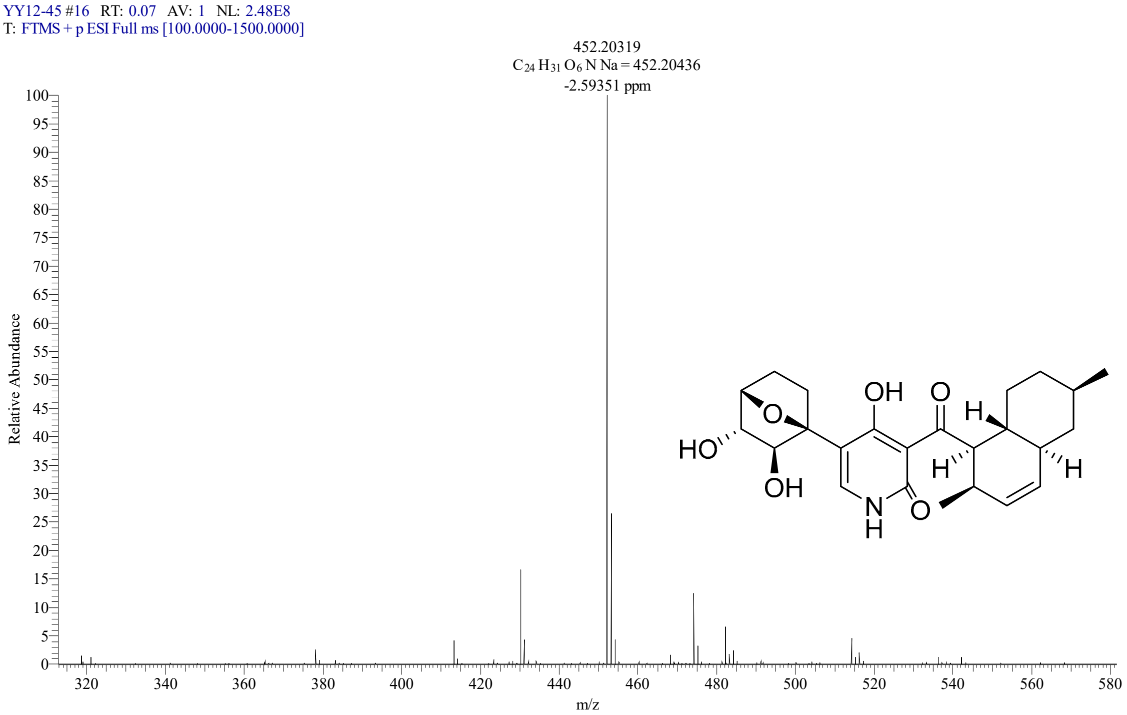

Figure S12. ^{1}H NMR (600MHz, DMSO-d_6) of **2**

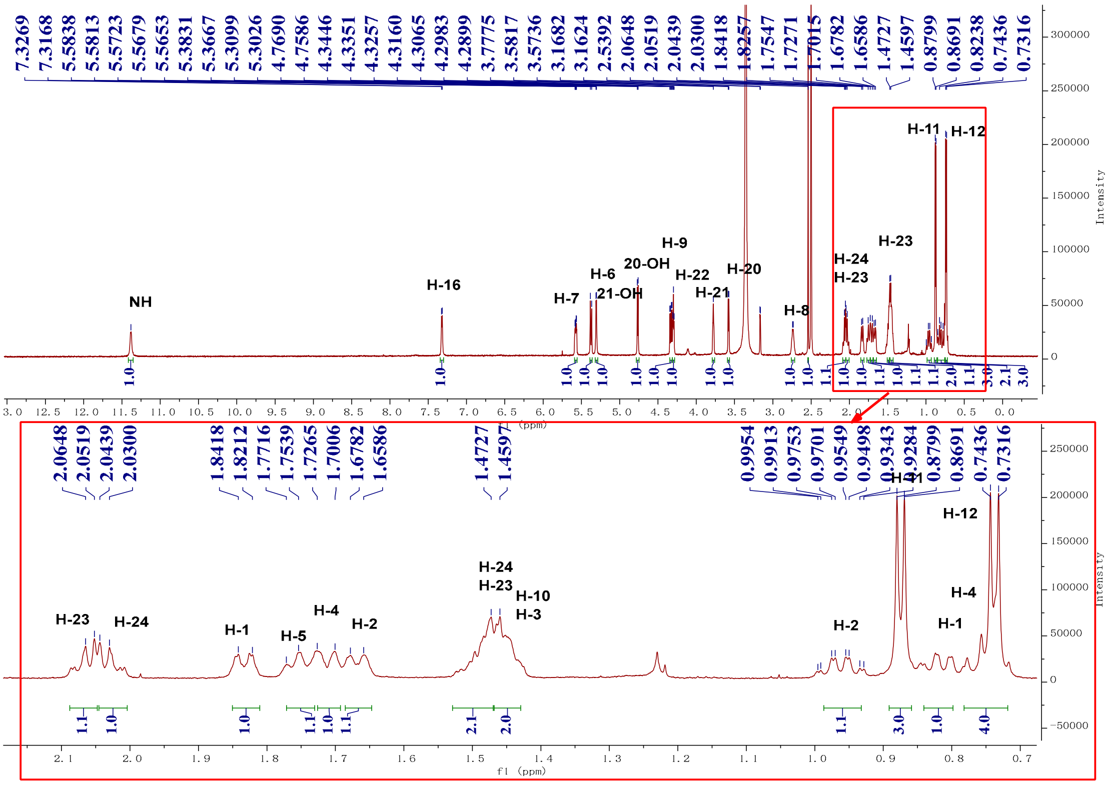

Figure S13. ^{13}C NMR (150 MHz, DMSO-d_6) of **2**

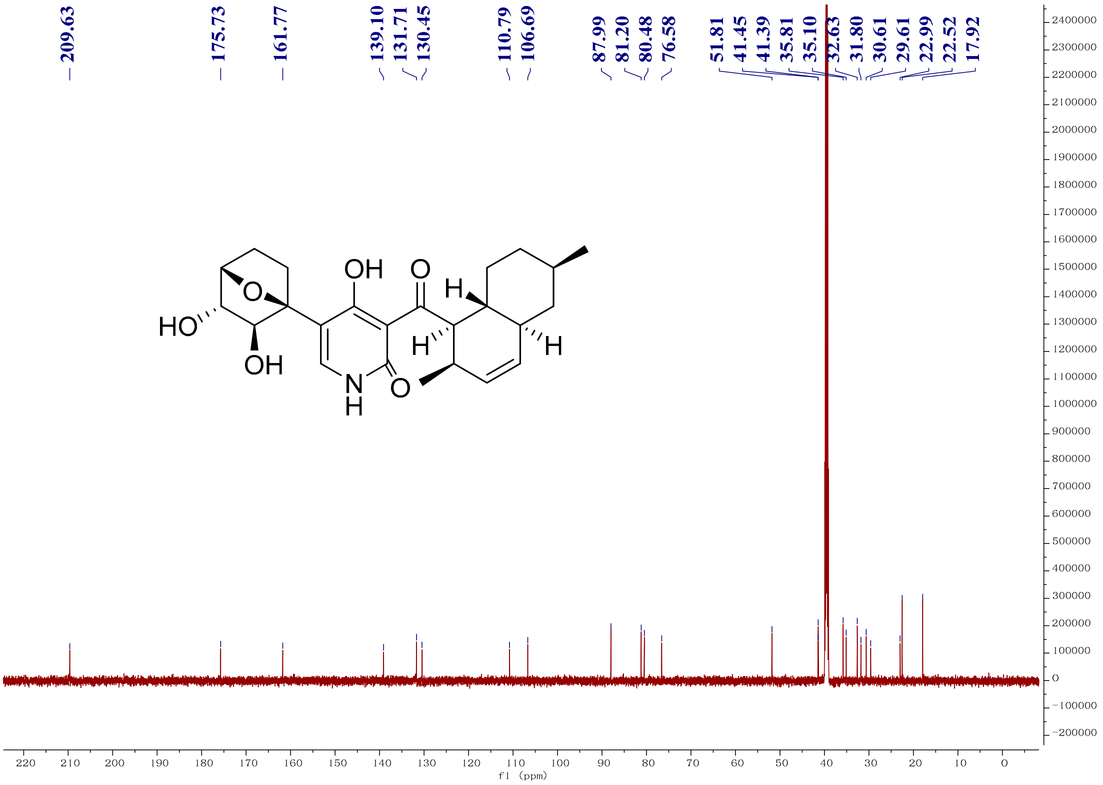

Figure S14. HSQC spectrum (DMSO-*d*6) of **2**

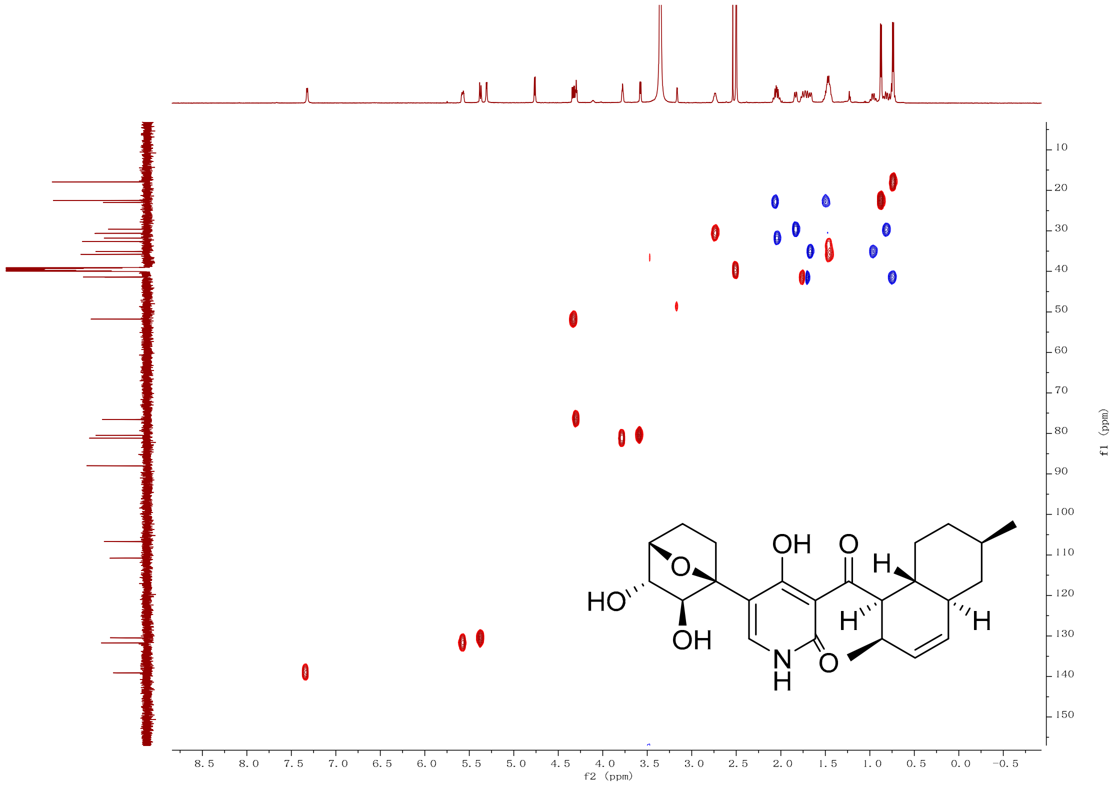

Figure S15. HMBC spectrum (DMSO-*d*6) of **2**

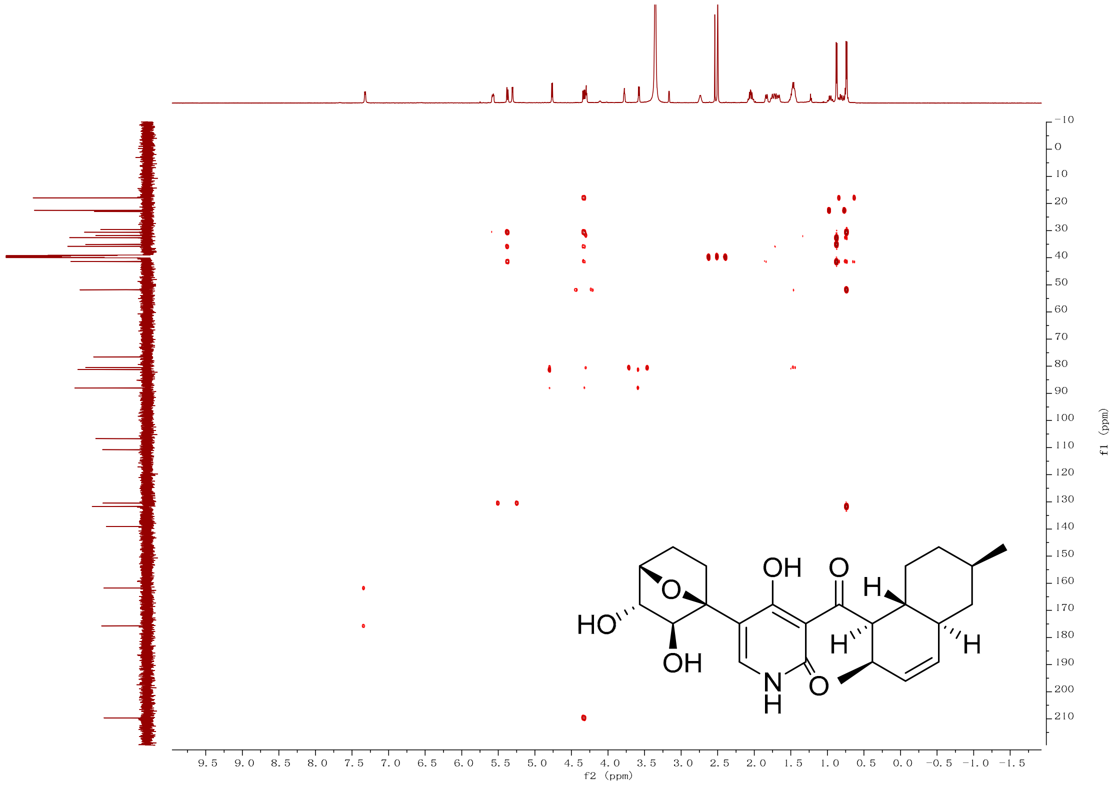

Figure S16. ^{1}H-^{1}H COSY spectrum (DMSO-d_6) of **2**

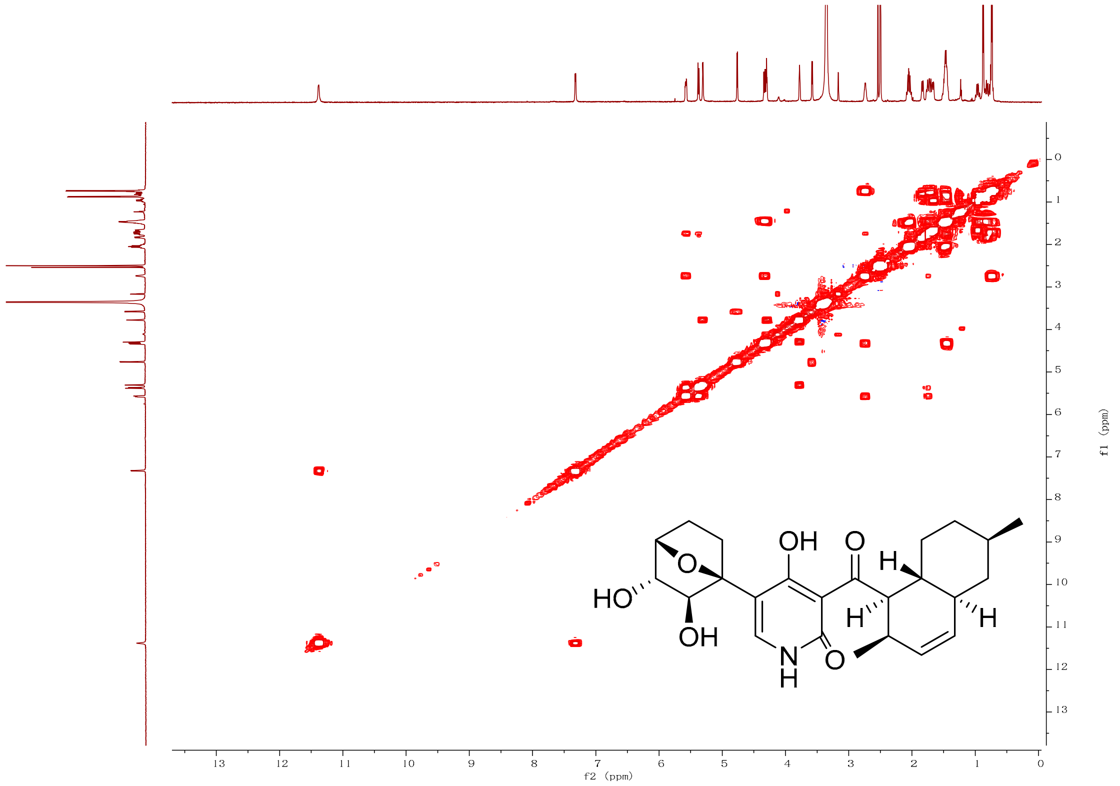

Figure S17. NOESY spectrum (DMSO-d_6) of **2**

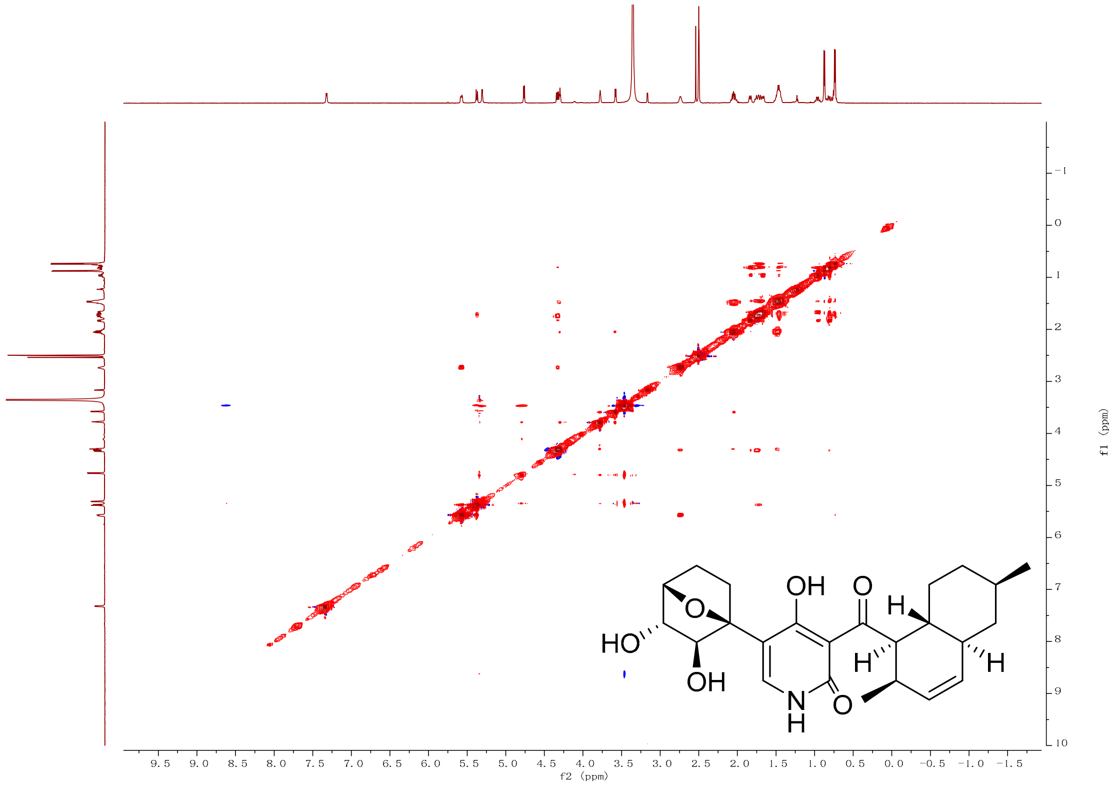

Figure S18. ^{1}H NMR spectrum (600MHz, Methanol-*d*$_4$) of **3**

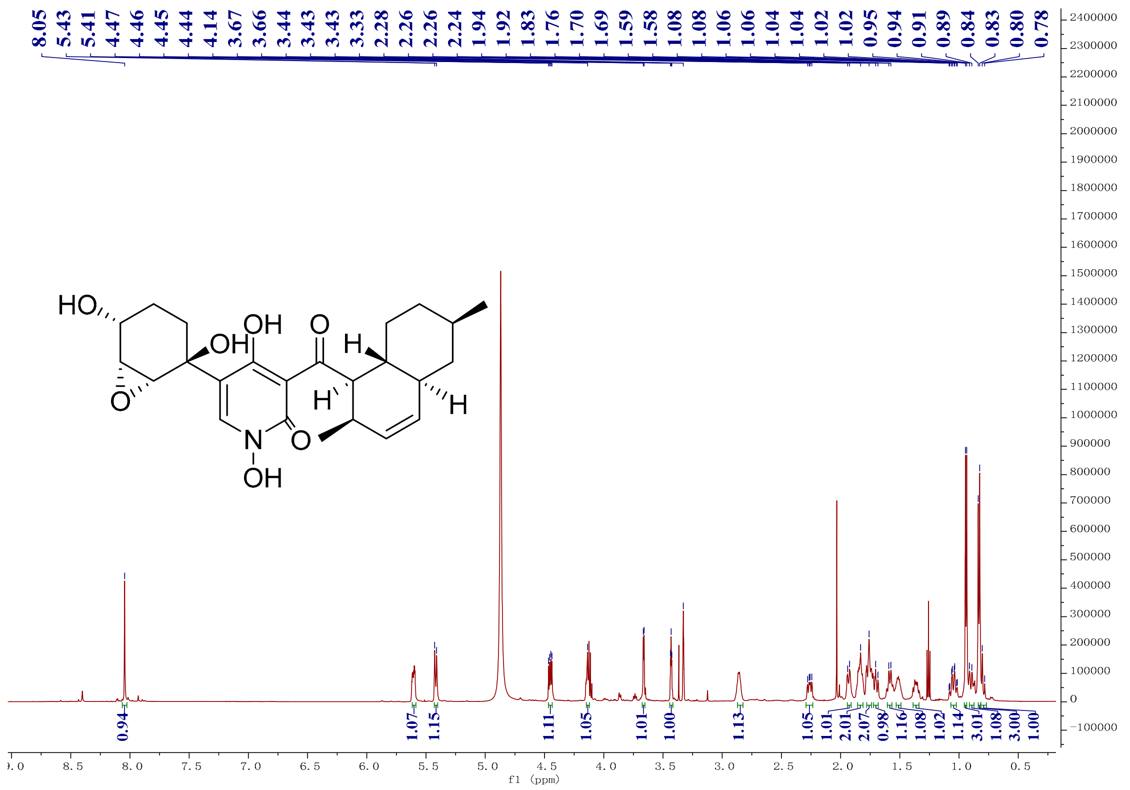

Figure S19. ^{13}C NMR spectrum (150 MHz, Methanol-*d*4) of **3**

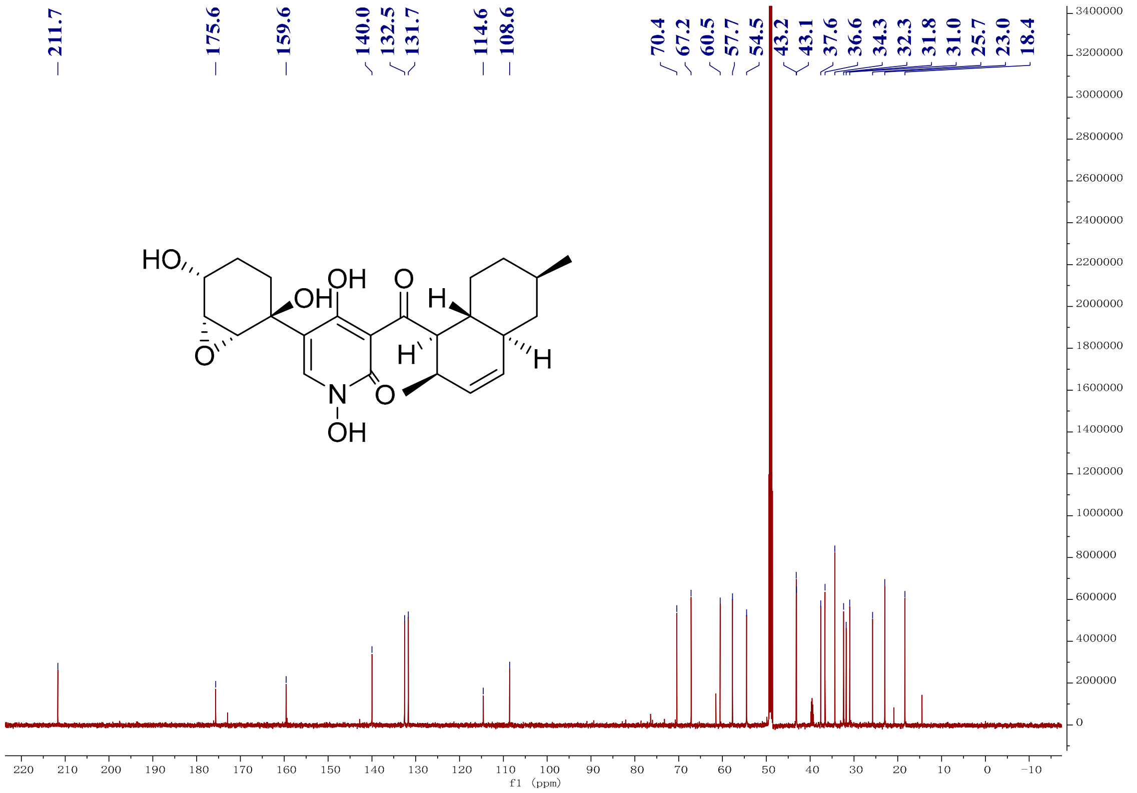

Figure S20. ^{1}H NMR spectrum (600MHz, Methanol-*d*4) of **4**

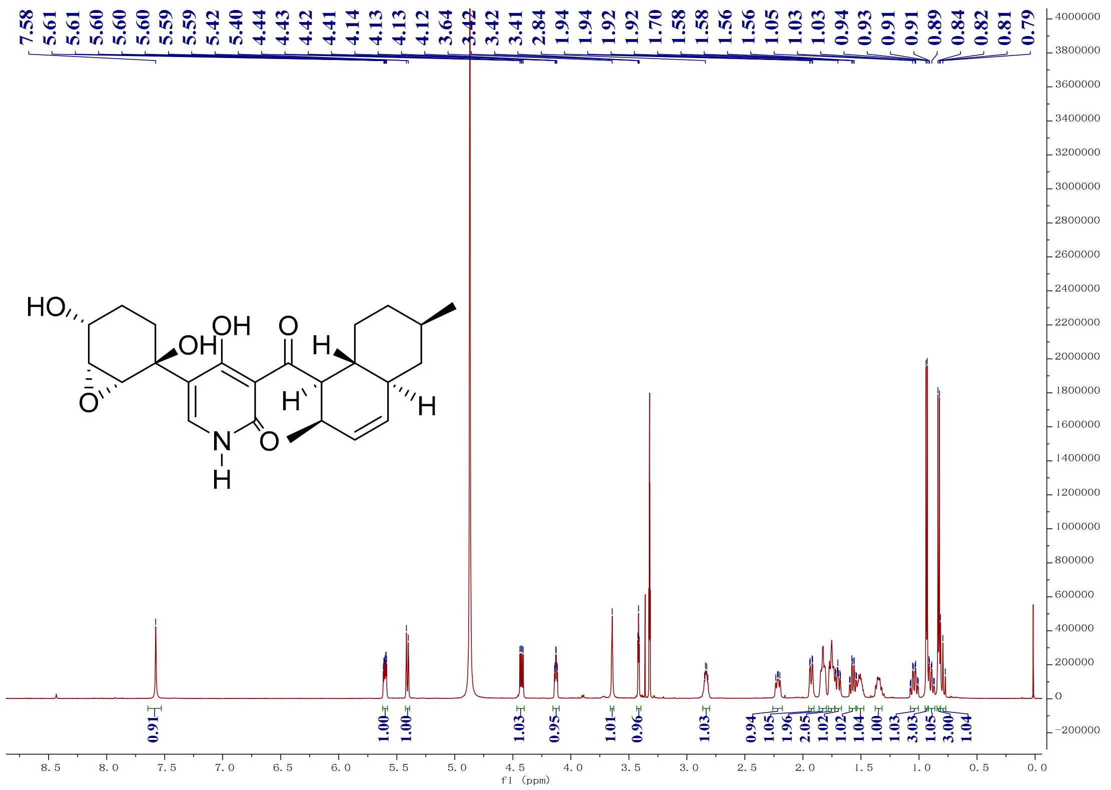

Figure S21. ^{13}C NMR spectrum (150 MHz, Methanol-*d*4) of **4**

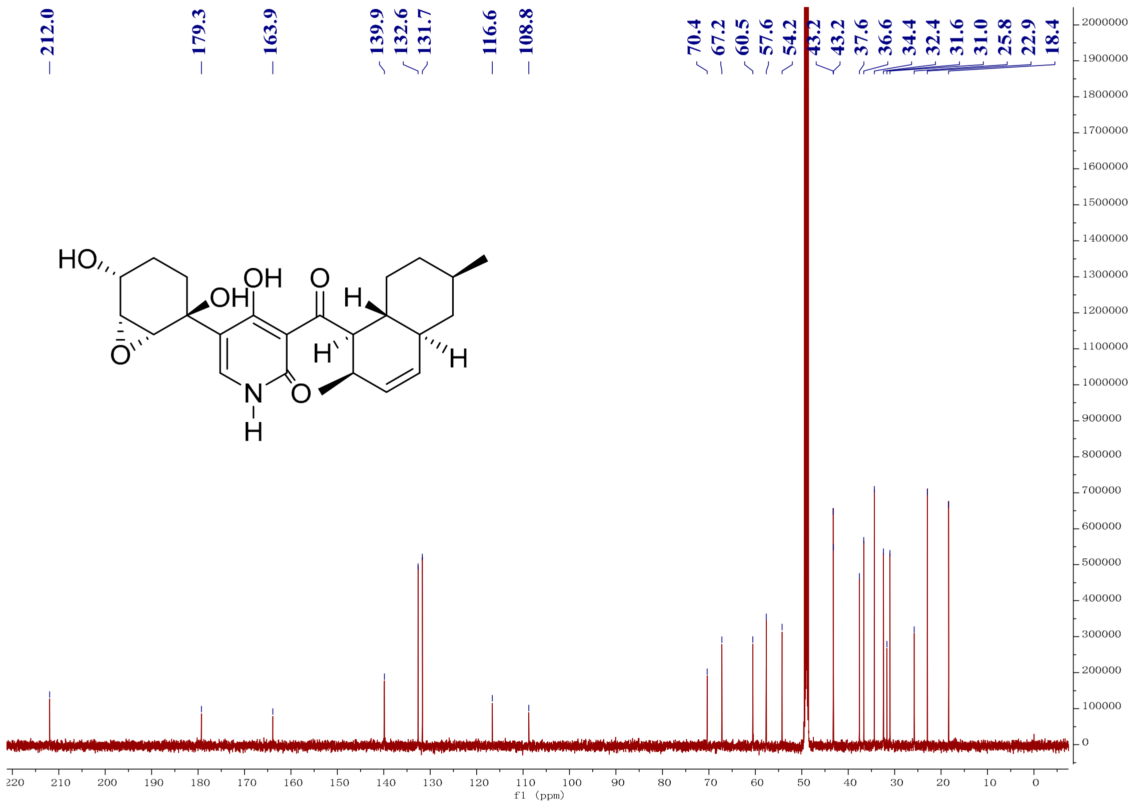

Supplement: Supplementary file 1 [file molecules-28-02192-s001.zip › molecules-2226772-supplementary.pdf]
